# Hormonal contraceptive use and prevalence of premenstrual symptoms in a multiethnic Canadian population

**DOI:** 10.1186/s12905-017-0450-7

**Published:** 2017-09-26

**Authors:** Alicia Caroline Jarosz, Joseph Jamnik, Ahmed El-Sohemy

**Affiliations:** Department of Nutritional Sciences, University of Toronto, Room 350, 150 College St, Toronto, ON M5S 3E2 Canada

**Keywords:** Premenstrual symptoms, Prevalence, Ethnicity, Hormonal contraceptives

## Abstract

**Background:**

Hormonal contraceptive use may be associated with a reduction in some premenstrual symptoms, however, the evidence remains equivocal. The objectives of the present study were to investigate the associations between ethnicity and hormonal contraceptive use with premenstrual symptoms.

**Methods:**

One thousand one hundred two women participating in the Toronto Nutrigenomics and Health Study provided data on their premenstrual symptoms and hormonal contraceptive use. Severity of symptoms was classified as none, mild, moderate, or severe. Prevalence of premenstrual symptoms was determined in the total population and among major ethnic groups. Logistic regressions were used to determine the association between ethnicity and prevalence of premenstrual symptoms. Logistic regressions were used to determine the associations between hormonal contraceptive use, and premenstrual symptoms, adjusting for ethnicity and other covariates.

**Results:**

Prevalence of individual symptoms varied, and the most commonly reported were cramps (75%), bloating (75%), mood swings (73%), increased appetite (64%), and acne (62%). Prevalence of cramps differed between ethnic groups with East Asians reporting a lower prevalence than Caucasians and South Asians (*p* < 0.05). Use of hormonal contraceptives was associated with a lower RR (95% CI) of experiencing moderate/severe: cramps (0.82, 0.72-0.93), clumsiness (0.22, 0.07-0.73), confusion (0.22, 0.09-0.54) and desire to be alone (0.45, 0.28-0.73). Hormonal contraceptive use was not associated with the risk of premenstrual symptoms at mild severity. Hormonal contraceptive use was not associated with symptoms of anxiety, bloating, mood swings, increased appetite, acne, fatigue, sexual desire, depression, nausea, headache and insomnia.

**Conclusion:**

This study demonstrates that East Asians may be at a lower risk of experiencing premenstrual cramps and that hormonal contraceptive use is associated with a lower risk of experiencing many, but not all, premenstrual symptoms at moderate/severe severity.

## Background

Premenstrual symptoms include a wide range of physical, psychological and behavioral symptoms, which occur in the late luteal phase of a woman’s reproductive cycle and subside a few days following the onset of menses [1]. Many symptoms have been described to date, and a few most commonly experienced somatic symptoms are bloating, headache, fatigue, and muscle cramps. Behavioural and psychological symptoms are also commonly experienced, such as anxiety, mood swings, changes in appetite, and depression [2, 3]. It is estimated that more than 80% of women regularly experience premenstrual symptoms, however, prevalence varies between studies and populations [4–10]. It is generally accepted that the prevalence is influenced by factors such as body weight and age, however, the association with ethnicity has been inconsistent [11, 12].

Little is known about the pathophysiology of premenstrual symptoms, and consequently, few effective therapies have been developed for them [1]. Due to the timing of the symptoms, changes in plasma levels of progesterone and estradiol are thought to be involved in their etiology [1]. Stabilizing fluctuations of these hormones during the luteal phase with the use of hormonal contraceptives may be effective in treating premenstrual symptoms [1], but the evidence has been inconsistent [1, 2].

Due to the large variation in frequencies of reported symptoms and their possible associations with ethnicity and hormonal contraceptive use, the objectives of this study were to determine the prevalence of various premenstrual symptoms in a multiethnic Canadian population and to assess their associations with hormonal contraceptive use.

## Methods

### Study sample

Subjects included 1636 men and women aged 20-29 years who participated in the Toronto Nutrigenomics and Health (TNH) study, which is a cross-sectional examination of young adults investigating genetics, lifestyle, and biomarkers of health [13, 14]. Recruitment occurred between 2004 and 2010. Participants provided overnight fasting blood samples and completed a general health and lifestyle questionnaire (GHLQ) which included questions regarding premenstrual symptoms, hormonal contraceptive (HC) use, and physical activity. Exclusion criteria included current pregnancy or breastfeeding. The study protocol was approved by the Ethics Review Board of the University of Toronto and participants provided written informed consent.

From the initial 1636 subjects, 520 men were excluded, 10 subjects were excluded due to missing GHLQ information, and 4 were excluded for lack of blood samples. The remaining 1102 female subjects were categorized into four ethnic groups based on self-reported ethnic status: Caucasian (*n* = 514), East Asian (*n* = 401), South Asian (*n* = 105), or Other (*n* = 82), as described previously [15]. Caucasians included those self-reported as European, Middle Eastern, or Hispanic. East Asians consisted of Chinese, Japanese, Korean, Filipino, Vietnamese, Thai, and Cambodian. South Asians included Bangladeshi, Indian, Pakistani, and Sri Lankan. The Other category included self-reported ethnicities of Aboriginal Canadians, Afro-Caribbeans, and those who self-reported belonging to ≥2 ethnic groups not included in the same category.

### GHLQ

Hormonal contraceptive use was self-reported in the GHLQ. Subjects were categorized as HC users (*n* = 320) and non-users (*n* = 782). HC users included subjects indicating current use of HCs, regardless of HC type or delivery method (transdermal, oral, vaginal, injection, etc.). Subjects also reported use of any medications in the past month. Use of anti-depressants, analgesics, or anxiolytics was considered in the present study as ‘PMS medication use’, due to their effects on premenstrual symptoms.

### Anthropometrics and physical activity

Subjects’ height and weight were measured using the protocol previously described by Garcia-Bailo et al. (2012) [16]. Subjects wore light clothing and removed their shoes during the measurements. Body mass index (BMI) was subsequently calculated in kg/m^2^. Subjects self-reported their physical activity in the GHLQ by estimating the amount of time they spent sleeping and engaging in light, moderate, and vigorous activity. Values were then converted into metabolic equivalent (MET) levels.

### Plasma samples and vitamin D measurement

Participants provided blood samples following a minimum 12-h overnight fast. Participants experiencing a temporary inflammatory condition (including a recent piercing or tattoo, acupuncture, a medical or dental procedure, a vaccination or immunization, flu, an infection, or a fever) underwent a two-week recovery period prior to providing blood samples. Samples were collected at LifeLabs Medical Laboratory Services (Toronto, Ontario, Canada), and 25-hydroxyvitamin D (25(OH)D) levels were measured at the University Health Network Specialty Lab at Toronto General Hospital (Toronto, Ont., Canada). Plasma 25(OH)D was measured by high-performance liquid chromatography–tandem mass spectrometry.

### Premenstrual symptoms

Premenstrual symptoms and severities were self-reported in a questionnaire included in the GHLQ. The questionnaire included the following symptoms: cramps; bloating/swelling/breast tenderness; mood swings/irritability/angry outbursts; increased appetite/food cravings; acne; sexual desire/activity change; fatigue; anxiety/tension/nervousness; depression; desire to be alone; confusion/difficulty concentrating/forgetfulness; nausea; insomnia; headache; and clumsiness. Symptom severities were classified as none, mild, moderate, or severe. Subjects were asked to indicate the severity at which they experienced each symptom, within the 5 days before the onset of their period and ending by the 4th day of their period. Subjects could also list other premenstrual symptoms experienced, however, due to the scarcity of other symptoms they were not included in the analyses. The premenstrual questionnaire was self-developed for the TNH study based on commonly reported symptoms and previously validated questionnaires.

### Statistical analysis

All statistical analyses were conducted using SAS (version 9.4; SAS Institute Inc., Cary, NC, USA). The α was set at 0.05 and all reported *p*-values are 2-sided. Subject characteristics were compared between HC users and non-users by chi-square analysis for categorical variables and t-tests for continuous variables. Distribution of continuous variables was assessed prior to analysis and log-transformed BMI was used in all subsequent analyses. Crude mean BMI values were reported for ease of interpretation.

The prevalence of premenstrual symptoms was defined as the frequency of subjects experiencing the symptoms at any severity (mild, moderate, or severe). Prevalence was calculated for each symptom in the total population, and separately for the major ethnic groups: Caucasians (*n* = 514), East Asians (*n* = 401), South Asians (*n* = 105), and Other (*n* = 82). Logistic regressions were used to determine differences in the prevalence of symptoms between the four ethnic groups. *P*-values were calculated in both unadjusted models as well as adjusted models which included the following covariates: age, BMI, HC use, physical activity, PMS medication use and plasma 25(OH)D concentrations. Benjamini-Yekutieli adjustments for multiple comparisons were applied (15 tests, α = 0.05: *p* < 0.015). Differences in the prevalence of each symptom between each pair of ethnic groups were also examined (Caucasians vs East Asians; Caucasians vs South Asians; Caucasians vs Other; East Asians vs South Asians; East Asians vs Other; South Asians vs Other) using logistic regressions.

Logistic regressions were used to examine the associations between HC use and premenstrual symptom severities. The proc. genmod procedure was conducted with a binomial distribution and a log link function. Moderate and severe symptom severities were combined due to the small number of subjects reporting severe symptoms. Relative risks (RR) and 95% confidence intervals (CI) were reported for associations between HC use and premenstrual symptoms. Univariate models were first used in Model 1, followed by multivariate models in Model 2 which adjusted for ethnicity, BMI, physical activity, PMS medication use and age. Covariates were selected based on their associations with HC use or premenstrual symptoms in the TNH study population and previous studies. Benjamini-Yekutieli adjustments for multiple comparisons were applied (30 tests, α = 0.05: *p* < 0.013).

## Results

### Study sample

Subject characteristics are shown in Table 1. The mean age of participants was 22.6 years. HC users were on average older (23 years) than non-users (22.4 years) (*p* = 0.0006). HC use differed between ethnic groups, with Caucasian women reporting the greatest use of HCs (43%), followed by Other (34%), South Asians (17%) and East Asians (13%). Reported physical activity was greater for HC users (8.1 met-hours/week) than non-users (7.5 met-hours/week) (*p* = 0.007). Log-transformed BMI and PMS medication use did not differ between HC users and non-users.

**Table 1:** Subject characteristics stratified by Hormonal Contraceptive (HC) use

	HC non-users (%)	HC users (%)	*p*-value
N	782	320	0.0006
Age (years)	22.4 ± 0.1	23.0 ± 0.1	
Ethnicity (%)			<0.0001
Caucasian	293 (57)	221 (43)	
East Asian	348 (87)	53 (13)	
South Asian	87 (83)	18 (17)	
Other	54 (66)	28 (34)	
Body mass index (kg/m^2^)*	22.4 ± 0.1	22.7 ± 0.2	0.11
Physical activity (met-h/wk)	7.5 ± 0.1	8.1 ± 0.2	0.007
PMS Medication Use	205 (26)	68 (21)	0.08

Shown are crude means and standard errors of continuous variables, and n (%) of categorical variables

*p*-values were obtained using chi-square tests for categorical variables and t-tests for continuous variables

*indicates log-transformed variable was used for obtaining *p*-value

### Prevalence of premenstrual symptoms

Prevalence of experiencing any premenstrual symptoms in the total population was 99%, and did not differ significantly between ethnic groups (*p* = 0.11). Prevalence of each premenstrual symptom in the total population and stratified by ethnicity is shown in Table 2. The most common symptoms experienced were cramps (75%), bloating (75%), mood swings (73%), increased appetite (64%), and acne (62%). Other premenstrual symptoms experienced were fatigue (55%), sexual desire (50%), anxiety (37%), desire to be alone (33%), depression (29%), headache (27%), confusion (21%), clumsiness (15%), nausea (15%), and insomnia (11%).

**Table 2:** Premenstrual symptom prevalence by ethnicity

Symptom	Total (%)	Caucasian (%)	East Asian (%)	South Asian (%)	Other (%)	Model 1	Model 2
	*N* = 1102	*N* = 514	*N* = 401	*N* = 105	*N* = 82	*p*-value	*p*-value
Cramps	75	79^a^	67^b^	84^a^	78^ab^	<0.0001	<0.0001
Bloating/Swelling/Breast Tenderness	75	79	70	70	78	0.01	0.16
Mood Swings/Irritability	73	73	72	74	67	0.68	0.68
Increased Appetite/Food Cravings	64	65	63	64	62	0.90	0.86
Acne	62	66	60	52	57	0.05	0.18
Fatigue	55	52	56	58	65	0.15	0.13
Sexual Desire/Activity Change	50	55	42	48	56	0.001	0.11
Anxiety/Tension/Nervousness	37	36	38	34	34	0.85	0.87
Desire to be alone	33	30	33	42	39	0.06	0.23
Depression	29	30	27	33	28	0.57	0.63
Headache	27	27	23	37	29	0.03	0.15
Confusion/Difficulty Concentrating/Forgetfulness	21	18	26	24	18	0.02	0.17
Clumsiness	15	14	18	15	11	0.26	0.27
Nausea	15	16	11	20	17	0.05	0.21
Insomnia	11	9	11	16	13	0.16	0.63

Sorted by total premenstrual symptom prevalence. Letters indicate prevalence values which differed significantly from each other in the adjusted model (*p* < 0.05)

In the unadjusted model symptom prevalence differed between ethnic groups for symptoms of cramps, bloating, sexual desire, headache, and confusion (*p* < 0.05). However, after adjustments for age, BMI, HC use, physical activity, medication use, and plasma 25(OH)D concentrations, only the prevalence of cramps differed between ethnic groups (*p* < 0.05). This association met adjustments for multiple comparisons (*p* < 0.015), where East Asians reported a lower prevalence of cramps than Caucasians and South Asians. Prevalence of bloating, mood swings, increased appetite, acne, fatigue, sexual desire, anxiety, desire to be alone, depression, headache, confusion, clumsiness, nausea, and insomnia did not differ between ethnic groups in the adjusted model.

### Premenstrual symptom associations with HC use

Associations between premenstrual symptoms and HC use are shown in Table 3. In the unadjusted model, HC use was associated with a lower risk of experiencing mild acne and the following symptoms at moderate/severe severity: cramps, fatigue, anxiety, clumsiness, confusion, depression, and desire to be alone. In Model 2, which adjusted for ethnicity, BMI, physical activity, PMS medication use and age, HC use was not associated with any symptoms at mild severity. HC use was associated with a lower RR (95% CI) of experiencing moderate/severe: cramps (0.82, 0.72-0.93), anxiety (0.63, 0.42-0.95), clumsiness (0.22, 0.07-0.73), confusion (0.22, 0.09-0.54), depression (0.55, 0.34-0.90), and desire to be alone (0.45, 0.28-0.73). Symptoms of cramps, clumsiness, confusion, and desire to be alone met Benjamini-Yekutieli criteria for multiple comparisons (30 tests, α = 0.05: *p* < 0.013). Premenstrual symptoms of bloating, mood swings, increased appetite, acne, fatigue, sexual desire, headache, nausea, and insomnia were not associated with HC use in Model 2. Low sample size precluded calculations of adjusted relative risks for moderate/severe nausea, where unadjusted RRs were: 0.53 (0.25, 1.13).

**Table 3:** Associations between HC use and premenstrual symptom severity

Symptom	Severity	HC non-users (%)	HC Users (%)	Model 1	Model 1	Model 2	Model 2
		*N* = 782	*N* = 320	Relative Risk	*p*-value	Relative Risk	*p*-value
Acne / Skin Blemish	None	310 (40)	111 (35)	REF		REF	
	Mild	318 (41)	161 (50)	1.17 (1.03,1.33)	0.02	1.12 (0.98,1.28)	0.10
	Moderate/Severe	154 (20)	48 (15)	0.91 (0.69,1.19)	0.49	0.84 (0.63,1.12)	0.23
Bloating / Swelling / Breast Tenderness	None	207 (26)	71 (22)	REF		REF	
	Mild	309 (40)	143 (45)	1.12 (0.99,1.26)	0.07	1.07 (0.95,1.22)	0.27
	Moderate/Severe	266 (34)	106 (33)	1.06 (0.92,1.23)	0.39	1.00 (0.86,1.16)	0.99
Cramps	None	185 (24)	89 (28)	REF		REF	
	Mild	256 (33)	128 (40)	1.02 (0.89,1.16)	0.82	0.93 (0.81,1.07)	0.29
	Moderate/Severe	341 (44)	103 (32)	0.83 (0.72,0.96)	0.01	0.82 (0.71,0.93)	0.002
Mood Swings / Crying Easily / Irritability /Angry Outbursts	None	212 (27)	92 (29)	REF		REF	
	Mild	287 (37)	128 (40)	1.01 (0.88,1.16)	0.87	1.02 (0.88,1.18)	0.80
	Moderate/Severe	283 (36)	100 (31)	0.91 (0.78,1.06)	0.24	0.89 (0.76,1.06)	0.19
Increased Appetite / Food Cravings	None	282 (36)	112 (35)	REF		REF	
	Mild	232 (30)	107 (33)	1.08 (0.91,1.28)	0.35	1.06 (0.89,1.27)	0.49
	Moderate/Severe	268 (34)	101 (32)	0.97 (0.82,1.15)	0.75	0.97 (0.82,1.16)	0.76
Fatigue	None	342 (44)	153 (48)	REF		REF	
	Mild	245 (31)	107 (33)	0.99 (0.83,1.17)	0.87	1.00 (0.83,1.20)	0.97
	Moderate/Severe	195 (25)	60 (19)	0.78 (0.61,0.99)	0.04	0.82 (0.64,1.05)	0.11
Headache	None	575 (74)	231 (72)	REF		REF	
	Mild	131 (17)	58 (18)	1.08 (0.82,1.43)	0.58	1.11 (0.84,1.49)	0.46
	Moderate/Severe	76 (10)	31 (10)	1.01 (0.68,1.50)	0.95	1.01 (0.67,1.51)	0.97
Anxiety / Tension / Nervousness	None	480 (61)	220 (69)	REF		REF	
	Mild	201 (26)	73 (23)	0.84 (0.67,1.06)	0.15	0.88 (0.69,1.13)	0.31
	Moderate/Severe	101 (13)	27 (8)	0.63 (0.42,0.94)	0.02	0.63 (0.42,0.95)	0.03
Clumsiness	None	655 (84)	278 (87)	REF		REF	
	Mild	90 (12)	39 (12)	1.02 (0.72,1.45)	0.92	1.07 (0.74,1.56)	0.71
	Moderate/Severe	37 (5)	3 (1)	0.20 (0.06,0.64)	0.007	0.22 (0.07,0.73)	0.01
Confusion / Difficulty Concentrating / Forgetfulness	None	599 (77)	267 (83)	REF		REF	
	Mild	121 (15)	48 (15)	0.91 (0.67,1.23)	0.53	1.00 (0.72,1.38)	1.00
	Moderate/Severe	62 (8)	5 (2)	0.20 (0.08,0.48)	0.0004	0.22 (0.09,0.54)	0.001
Sexual Desire / Activity Change	None	407 (52)	147 (46)	REF		REF	
	Mild	233 (30)	112 (35)	1.19 (1.00,1.41)	0.05	1.14 (0.95,1.37)	0.16
	Moderate/Severe	142 (18)	61 (19)	1.13 (0.88,1.46)	0.33	0.96 (0.74,1.23)	0.74
Insomnia	None	688 (88)	293 (92)	REF		REF	
	Mild	75 (10)	25 (8)	0.80 (0.52,1.23)	0.31	0.91 (0.57,1.43)	0.68
	Moderate/Severe	19 (2)	2 (1)	0.25 (0.06,1.08)	0.06	0.23 (0.05,0.99)	0.05
Nausea^a^	None	664 (85)	277 (87)	REF		REF	
	Mild	81 (10)	35 (10)	1.03 (0.71,1.50)	0.87	0.95 (0.64,1.41)	0.80
	Moderate/Severe	37 (5)	8 (3)	0.53 (0.25,1.13)	0.10	N/A	N/A
Depression	None	543 (69)	236 (74)	REF		REF	
	Mild	150 (19)	65 (20)	1.00 (0.77,1.29)	0.99	0.96 (0.73,1.26)	0.77
	Moderate/Severe	89 (11)	19 (6)	0.53 (0.33,0.85)	0.008	0.55 (0.34,0.90)	0.02
Desire to be alone	None	499 (64)	238 (75)	REF		REF	
	Mild	180 (23)	62 (19)	0.78 (0.60,1.01)	0.06	0.80 (0.62,1.04)	0.10
	Moderate/Severe	103 (13)	19 (6)	0.43 (0.27,0.69)	0.0004	0.45 (0.28,0.73)	0.001

Model 1 contains unadjusted relative risks and *p*-values

Model 2 contains relative risks and *p*-values adjusted for ethnicity, log-transformed BMI, physical activity, age, and medication use

^a^indicates no values obtained in Model 2 due to low sample size

## Discussion

In this study, we investigated the prevalence of 15 common premenstrual symptoms and their associations with hormonal contraceptive use in a multiethnic population of young adults living in Canada. Our findings show that the prevalence of individual premenstrual symptoms varies widely between the symptoms, and we observed ethnic differences in the prevalence of cramps. We also found that HC use was associated with a lower risk of experiencing several, but not all, premenstrual symptoms at moderate/severe severity. No associations were observed between HC use and the risk of experiencing mild premenstrual symptoms.

In our population 99% of the subjects reported experiencing premenstrual symptoms. The same prevalence estimates were found in female university students in Thailand and Iran [8, 9]. Prevalence reported in other studies have been slightly lower and have ranged from 80% to 95% [4–7, 17]. These variations in prevalence estimates may be explained by differences in symptom assessment, subject population, and subject characteristics such as age [18]. For example, the lowest prevalence of 80% was reported in a German community survey which included adolescent subjects aged 14–24 years [6]. The inclusion of adolescents could explain the lower prevalence, as was shown in a previous study which found that subjects under 20 or over 45 years of age had the lowest symptom prevalence, with prevalence peaking at age 35 [10]. Alternatively, a survey of only married Iranian women from health clinics aged 20–45 reported a prevalence of 86% [17]. Two previous studies that included women of similar age as in the present study reported similar prevalence for the various premenstrual symptoms [8, 9].

The most commonly experienced symptoms in the present study were cramps (75%), bloating (75%), irritability (73%), increased appetite (64%), and acne (62%). These differed from those reported in other studies, and as expected, investigations into the nature of the most commonly experienced symptoms have yielded varying results depending on the population studied [17, 19, 20]. In a recent survey of Iranian women, the most common symptoms reported were tiredness (70%), backache (68%), headache (59%), and insomnia (50%) [17]. The most common premenstrual symptoms reported in a population of Turkish medical students were bloating (90%), irritability (88%), breast tenderness (83%), and anxiety (74%) [19]. However, a study involving a Mexican population demonstrated abdominal cramping to be the most prevalent symptom (54%), while only 8% of women reported irritability [20]. Discrepancies in the prevalence of symptoms may be explained by several factors including variations in premenstrual symptom questionnaires, BMI, age, cultural factors, and environmental exposures. The questionnaire used in the present study differed from those used by others [17, 19, 20], which could account for some of the variation in symptom reporting. For example, the questionnaire used by Goker et al. did not include symptoms of acne, appetite changes, or cramps which were among the five most commonly experienced symptoms in the present population [19].

The effect of ethnicity in relation to premenstrual symptoms remains controversial. Sternfeld et al. showed that relative to Whites, Hispanics reported a greater severity of premenstrual symptoms whereas Asians reported a lesser severity [12]. Several studies involving US populations have shown significant differences in symptom prevalence between White and Black women, but these racial differences were not present for all symptoms [21–23]. This is in line with the results of the present study which revealed ethnic differences in the prevalence of some, but not all, symptoms and no ethnic differences in the total prevalence. In the present study, many symptoms were observed to differ by ethnicity in our unadjusted models but after adjustments for potential confounding variables these differences were no longer significant. Following adjustments, ethnic differences in prevalence were observed only for cramps which met adjustments for multiple comparisons. East Asian participants reported a lower prevalence of cramps compared to Caucasian and South Asian participants. Although this may reflect differences in genetics or cultural factors that may put East Asians at lesser risk of some premenstrual symptoms, it could also be explained by cultural differences in the interpretation and reporting of symptoms [24]. Ethnic differences in premenstrual symptom reporting have been previously observed and it was suggested that differences in the clustering of symptoms in Chinese women compared to Western women may be a result of differences in the conceptualization of the integration of organ systems and their relation to health and disease influenced by Traditional Chinese Medicine [24]. Nonetheless, these findings may guide researchers and healthcare practitioners in determining high-risk populations for premenstrual symptoms, and should be supported by future large-scale studies on Canadian populations.

In the present study, hormonal contraceptive use was associated with a lower risk of experiencing moderate/severe cramps, desire to be alone, clumsiness and confusion. Use of hormonal contraceptives was not associated with mild premenstrual symptoms. These findings are in agreement with three previous studies that found a decrease in the overall prevalence of symptoms as well as a decrease in the number and severity of emotional symptoms in women using oral contraceptives [12, 25, 26]. Two studies found no association between HC use and premenstrual symptoms [10, 27]. One study sampling 400 Iranian women observed a greater prevalence of several premenstrual symptoms in HC users versus non-users [17]. These studies, however, assessed the effects of HC use on grouped symptom prevalence and severity, while the present study identified specific premenstrual symptoms and severities which are associated with HC use. Grouping of symptoms likely accounted for these discrepancies in findings of associations between HC use and premenstrual symptoms. As shown in the present study, not all symptoms are associated with HC use and including their prevalence likely impacted previous findings. The present findings emphasize the importance of examining individual premenstrual symptoms in research investigating the efficacy of treatments for premenstrual symptoms.

The observed improvement of premenstrual symptoms with HC use has largely been attributed to stabilizing ovarian sex steroid fluctuations during the reproductive cycle [28]. Treatments preventing ovulation, such as long-acting GnRH agonists and bilateral oophorectomy, have been highly effective in diminishing premenstrual symptoms [28]. HCs may present a more favorable option for the management of premenstrual symptoms as they are accompanied by far fewer and less severe side effects [1]. Some HCs also possess anti-aldosterone and anti-androgenic properties that likely enhance their effects on premenstrual symptoms [1, 29]. There is some evidence that the effect of HC use on premenstrual symptoms is dependent on the HC formulation and regimen [30, 31]. In the present study, sample size limitations precluded the ability to study the effects of different HC formulations on premenstrual symptoms. Interestingly, HC use is associated with a higher concentration of pro-inflammatory proteins [32] which have also been linked to an increase in the severity of some premenstrual symptoms [33, 34].

The present study has some limitations. The questionnaire did not specify a retrospective time-frame for experiencing the listed premenstrual symptoms, which may have resulted in an under-reporting or over-reporting of symptoms depending on the participant’s interpretation of the question. The present study relied on retrospective symptom reporting which may result in an over-reporting of premenstrual symptoms [35]. This likely did not impact the associations with HC use, as ethnicity was adjusted for in that analysis and there is no evidence that symptom over-reporting would be more common in HC users than non-users.

## Conclusions

This cross-sectional examination of a young multiethnic population of Canadian women shows that 99% of women experience some type of premenstrual symptom, while the prevalence of individual symptoms varies widely. Findings suggest that ethnicity is not a risk factor for experiencing most premenstrual symptoms, although East Asians may be at a lower risk of premenstrual cramps. Use of hormonal contraceptives may put women at a lower risk of experiencing some premenstrual symptoms, but this effect may be dependent on the nature and severity of the symptom.

## Abbreviations

25(OH)D: 25-Hydroxyvitamin D
BMI: Body mass index
CI: Confidence interval
GHLQ: General health and lifestyle questionnaire
HC: Hormonal contraceptive
MET: Metabolic equivalent
RR: Relative risk
TNH: Toronto Nutrigenomics and Health
